# PSY Inhibits Colorectal Cancer Growth and Exhibits an In Vitro Synergistic Interaction with 5-Fluorouracil

**DOI:** 10.3390/ijms27209238

**Published:** 2026-10-17

**Authors:** Sanghee Han, Hail Kim, Hyunjin Choi, Minseo Oh, Min Young Lee, Junhee Lee, In Jin Ha, Kwang Seok Ahn, Seok-Geun Lee

**Affiliations:** 1 Department of Biomedical Science and Technology, Kyung Hee University, Seoul 02447, Republic of Korea; sanghee8757@khu.ac.kr (S.H.); khi3598@khu.ac.kr (H.K.);; 2 Korean Medicine Clinical Trial Center, Kyung Hee University Korean Medicine Hospital, Seoul 02454, Republic of Korea; 3 College of Korean Medicine, Kyung Hee University, Seoul 02447, Republic of Korea; 4 BioNanocomposite Research Center, Kyung Hee University, Seoul 02447, Republic of Korea

**Keywords:** colorectal cancer, PSY, endoplasmic reticulum stress, lipid metabolism, 5-fluorouracil, drug combination

## Abstract

PSY is a three-herb formula comprising Patriniae Radix, Mori Cortex Radicis, and Coix Seed that was previously shown to inhibit STAT3 signaling in colorectal cancer (CRC) cells. Here, we investigated molecular and metabolic responses to PSY, its antitumor activity in an HCT116 xenograft model, and its interactions with chemotherapy in vitro. Representative chemical markers were qualitatively verified by UPLC-ESI-QTOF MS/MS. Exploratory RNA sequencing with two biological replicates per group highlighted stress-associated and metabolic processes for further investigation. PSY increased BiP, phosphorylated eIF2α, ATF4, and CHOP levels, intracellular reactive oxygen species (ROS) levels, and mitochondrial membrane depolarization, while reducing the phosphorylation of PI3K/AKT/mTOR- and ERK-associated proteins. PSY increased the p-AMPK/AMPK ratio and decreased the p-ACC/ACC ratio. PSY reduced several lipid-biosynthetic transcripts and decreased the relative abundance of selected fatty acids in xenograft tumors. Oral administration of PSY suppressed HCT116 xenograft growth without significant body-weight loss. Combination-index (CI) analysis indicated CI-defined in vitro synergistic interactions with 5-fluorouracil (5-FU) at multiple concentration pairs, with interaction patterns varying across HCT116, HT-29, and SW480 cells. Direct cell counting supported the combination response in HCT116 and SW480 cells. The combination increased apoptosis and reduced STAT3 and NF-κB p65 phosphorylation relative to single treatments. These findings characterize concurrent, partly discordant signaling and metabolic responses to PSY and support further preclinical evaluation of its in vitro interaction with 5-FU.

## 1. Introduction

Colorectal cancer (CRC) remains one of the most common malignancies worldwide and is driven by complex molecular alterations involving inflammatory signaling, oncogenic survival pathways, stress adaptation, metabolic reprogramming, and resistance to cytotoxic therapy [1,2]. STAT3 and NF-κB are major regulators of inflammation-associated tumor progression, whereas PI3K/AKT/mTOR and ERK signaling support CRC cell proliferation, survival, invasion, and therapeutic resistance [2,3,4,5]. Cancer cells also depend on adaptive stress responses and metabolic plasticity to maintain growth under unfavorable conditions. Endoplasmic reticulum (ER) stress and the unfolded protein response (UPR) may promote tumor-cell survival under moderate stress but contribute to cell death when stress becomes excessive or prolonged [6,7]. In parallel, altered lipid metabolism supports membrane biosynthesis, redox homeostasis, inflammatory mediator production, and tumor-cell survival and has emerged as an important metabolic vulnerability in CRC [5,8,9].

The molecular heterogeneity of CRC provides a rationale for therapeutic approaches capable of modulating multiple interconnected pathways [10,11,12]. Natural products and multi-component herbal preparations represent potential sources of such multi-target pharmacological activity [10,12]. Natural compounds have also been investigated as adjuncts to conventional CRC chemotherapy, with preclinical studies suggesting that selected polyphenols can modulate multiple signaling pathways associated with treatment response and chemoresistance [13]. However, their biological complexity also requires rigorous chemical characterization and mechanistic validation to distinguish reproducible pharmacological effects from nonspecific cytotoxicity and to establish their potential relevance to cancer therapy.

PSY is a three-herb formula composed of *Patriniae* Radix, *Mori Cortex* Radicis, and *Coix* Seed. It was developed by modifying the traditional prescription Paejangsan (敗醬散, *bài jiàng sǎn*) through the addition of *Mori Cortex* Radicis according to traditional prescription-modification principles [14,15,16,17]. In our previous study, PSY was chemically profiled and shown to inhibit CRC-cell proliferation and reduce STAT3 signaling [14]. The ethanol extract showed greater anti-CRC activity than the corresponding water extract, and phytochemical analysis identified 38 constituents belonging to several chemical classes, including organic acids, iridoids, lignans, prenylflavonoids, and fatty acids [14]. These findings established the chemical and pharmacological basis of PSY; however, its broader molecular and metabolic actions, in vivo antitumor activity, and interactions with standard CRC chemotherapeutic agents remained undefined.

The present study extends the characterization of PSY by integrating chemical marker verification, exploratory transcriptomic profiling, molecular and functional analyses, tumor lipid profiling, an HCT116 xenograft model, and drug-combination testing. We examined molecular and metabolic responses associated with PSY treatment and evaluated its interaction with 5-fluorouracil (5-FU) in vitro. The xenograft experiment evaluated the antitumor activity of PSY alone. Together these approaches characterize the activity of PSY in the models studied and inform further preclinical investigation of the PSY–5-FU combination.

## 2. Results

### 2.1. Verification of Representative Chemical Markers in PSY

**Figure 1:**
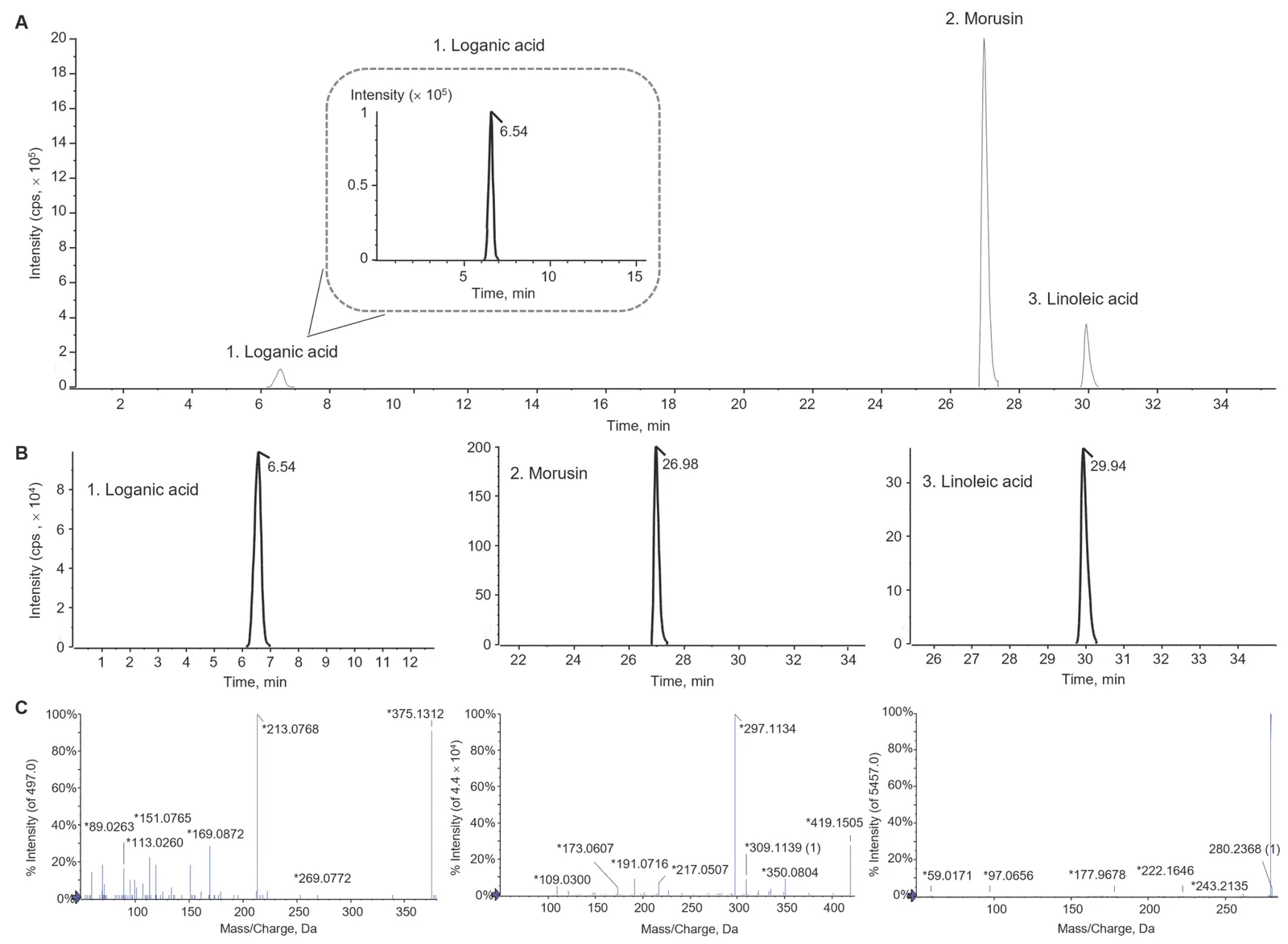
Verification of representative chemical markers in PSY by UPLC-ESI-QTOF MS/MS. Representative extracted ion chromatograms (XICs) of the PSY extract (**A**) and authentic reference standards (**B**), together with the corresponding MS/MS spectra (**C**), were obtained in negative-ion mode. Loganic acid (1), morusin (2), and linoleic acid (3) were verified as representative markers associated with *Patriniae* Radix, *Mori Cortex* Radicis, and *Coix* Seed, respectively. Chromatographic signal intensities are expressed as counts per second (cps) in panels (**A**,**B**). MS/MS spectra are shown as relative intensity (%) in panel (**C**). In panel (**C**), asterisks (*) preceding m/z values indicate peaks identified as monoisotopic by PeakView, whereas numbers in parentheses following m/z values indicate the estimated charge states.

To verify representative chemical markers in the PSY extract used for the pharmacological experiments, the extract was analyzed by UPLC-ESI-QTOF MS/MS. Extracted ion chromatograms showed peaks corresponding to loganic acid, morusin, and linoleic acid (Figure 1A). The chromatographic retention characteristics and MS/MS fragmentation patterns of these peaks were consistent with those of the corresponding authentic reference standards (Figure 1B,C). These compounds were selected as representative markers based on our previous profiling of PSY [14]. The analysis supported qualitative identification of the markers in the extract; their concentrations were not determined.

### 2.2. Exploratory Transcriptomic Profiling Identifies PSY-Responsive Molecular Networks in CRC Cells

**Figure 2:**
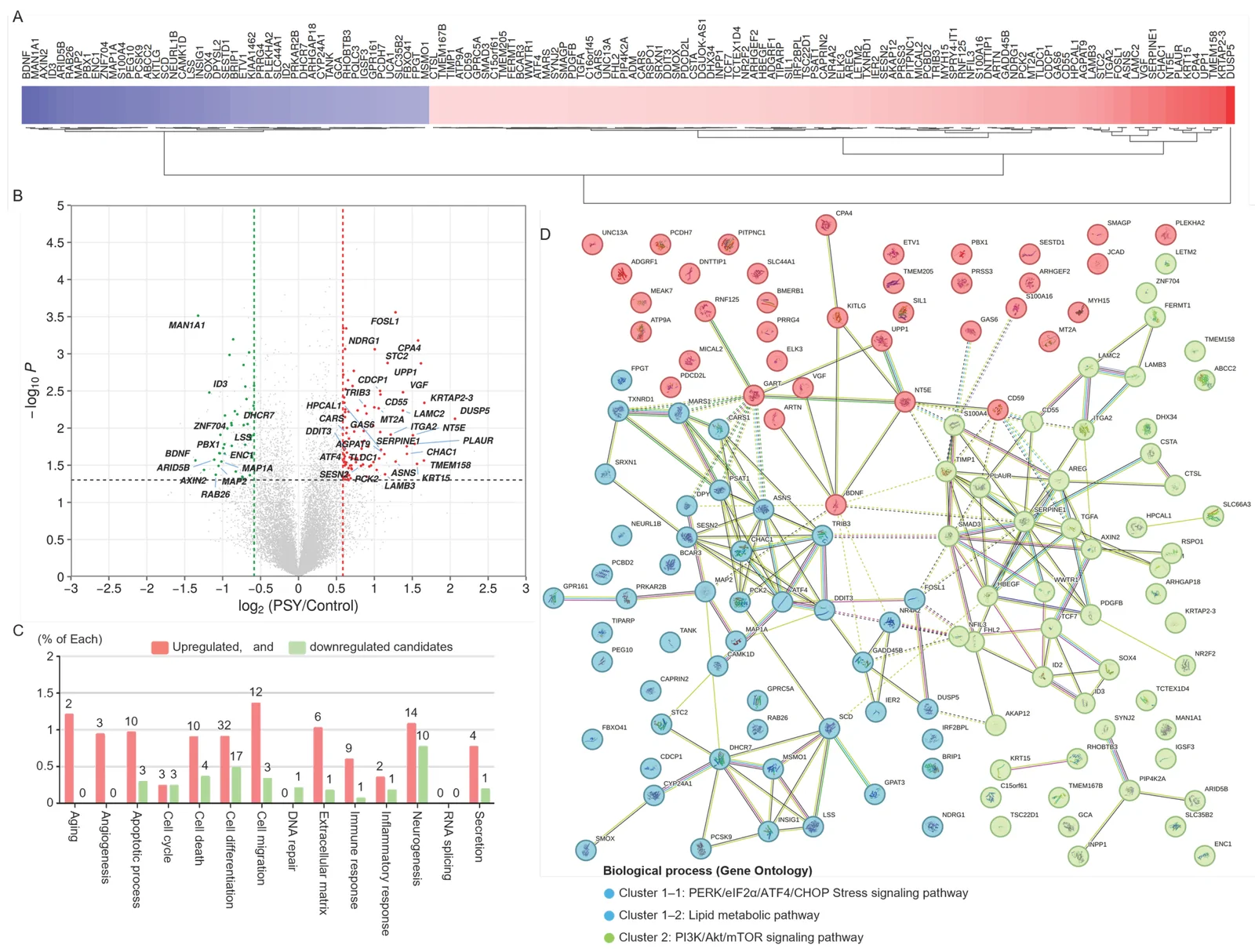
Exploratory transcriptomic profiling of PSY-treated HCT116 cells. Cells were treated with PSY (50 μg/mL) or vehicle for 24 h (*n* = 2 independent biological replicates per group). (**A**) Heatmap showing gene-expression fold changes (FC; PSY/control) for 140 exploratory candidates selected using a PSY/control ratio ≥ 1.5 or ≤ 2/3, unadjusted *p* < 0.05, and mean normalized log_2_ expression ≥ 4 in at least one group. White indicates no change (FC = 1), red indicates increased expression (FC > 1), and blue indicates decreased expression (FC < 1). (**B**) Volcano plot of the original analysis. The horizontal line indicates unadjusted *p* = 0.05, and the vertical lines indicate log_2_FC of ±log_2_(1.5). Red and green dots indicate genes meeting the exploratory candidate criteria with increased and decreased expression, respectively, whereas gray dots indicate genes that did not meet these criteria. No gene met BH-adjusted *p* < 0.05 in the original analysis. (**C**) Gene Ontology (GO) biological-process classification of the exploratory candidates. Pink and green indicate increased and decreased expression, respectively. (**D**) STRING network showing known and predicted functional association among proteins encoded by the exploratory candidates. Nodes colored according to the three highlighted GO-term clusters shown in the legend indicate proteins associated with those clusters. Red nodes indicate proteins not included in these three highlighted clusters but that may be associated with other GO terms. Icons within nodes indicate proteins for which known or predicted structural information is available in STRING. Solid lines indicate associations between proteins within the same cluster, whereas dashed lines indicate associations between proteins belonging to different clusters. Original and complementary DESeq2 statistics for the 140 exploratory candidates are provided in Supplementary Table S1A.

Exploratory RNA sequencing was performed using two independent biological replicates per group. Using the original criteria of a PSY/control expression ratio ≥ 1.5 or ≤2/3, an unadjusted *p* value < 0.05, and mean normalized log_2_ expression ≥ 4 in at least one group, 140 candidate genes were selected (93 increased and 47 decreased). The heatmap and volcano plot summarize expression changes in this exploratory analysis (Figure 2A,B). Benjamini–Hochberg (BH) adjustment across all 18,032 computable original tests identified no genes at adjusted *p* < 0.05 (minimum adjusted *p*, 0.1321).

A complementary DESeq2 analysis of the same raw-count dataset identified 262 genes at adjusted *p* < 0.05, without an additional fold-change cutoff. These included 108 of the original 140 candidates (77.1%); 119 candidates (85.0%) met adjusted *p* < 0.10. The direction of change agreed between the two analyses for all 140 candidates. Complete gene-level results, including raw counts, fold changes, raw *p* values, adjusted *p* values, and the original-candidate designation, are provided in Supplementary Table S1C. These findings remain hypothesis-generating given the limited biological replication.

Gene Ontology biological-process classification indicated that the identified genes were distributed across several processes relevant to tumor biology, including apoptosis and cell death, cell-cycle regulation, cell migration, extracellular matrix-related processes, immune and inflammatory responses, and RNA splicing (Figure 2C).

STRING-based PPI analysis revealed interconnected gene clusters associated with ER stress and the UPR, lipid metabolism, and PI3K/AKT/mTOR-related signaling (Figure 2D). These exploratory findings guided the selection of stress-associated and metabolic processes for subsequent molecular and cellular characterization.

### 2.3. PSY Alters Stress-Associated and Survival Signaling Markers and Induces Oxidative and Mitochondrial Responses in HCT116 Cells

**Figure 3:**
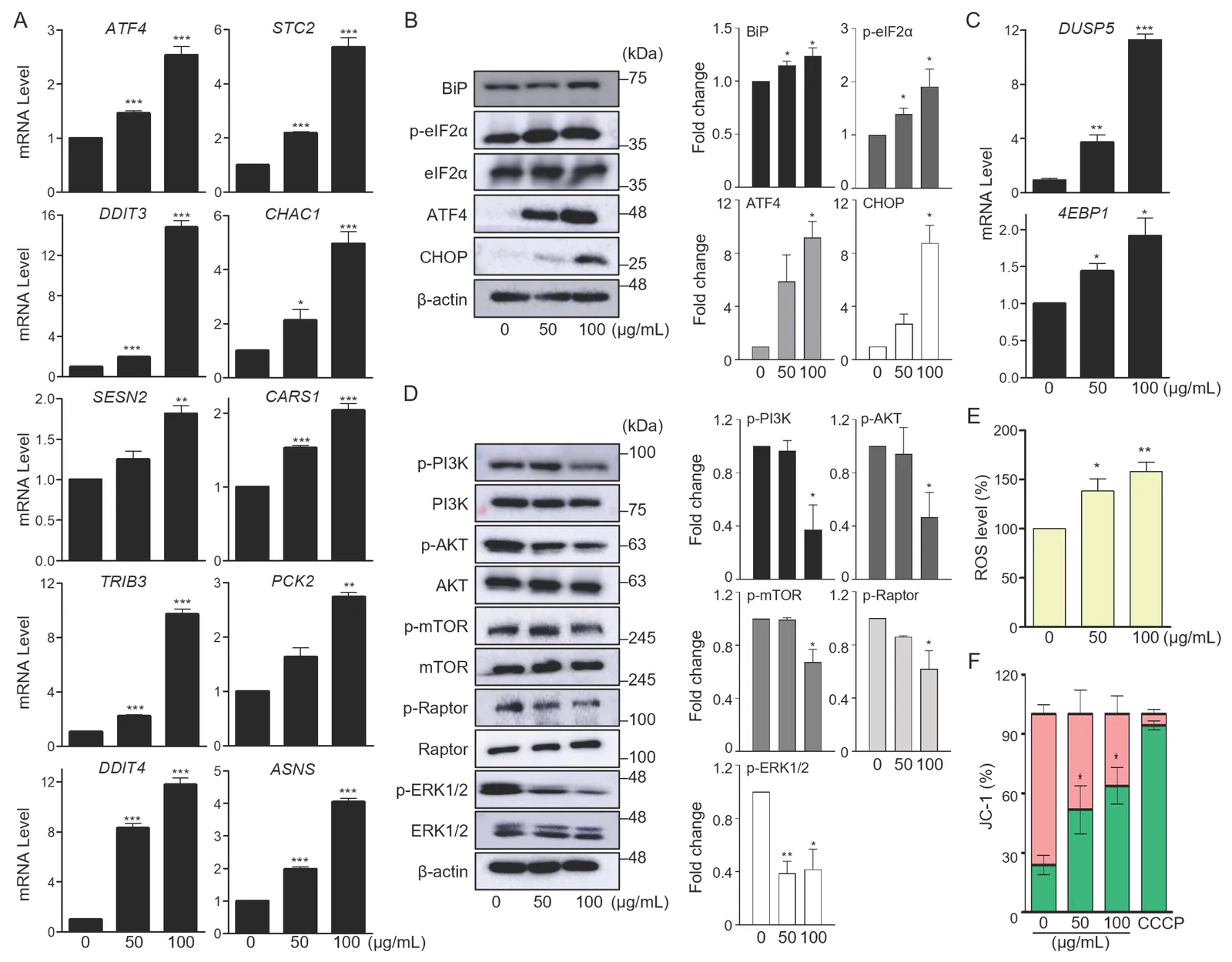
PSY alters stress-associated and survival-signaling markers and induces oxidative and mitochondrial responses in HCT116 cells. Cells were treated with PSY at the indicated concentrations. (**A**) Quantitative real-time (RT)-PCR analysis of genes associated with ER stress and the integrated stress response, including *ATF4*, *DDIT3*/*CHOP*, *SESN2*, *TRIB3*, *DDIT4*, *STC2*, *CHAC1*, *CARS1*, *PCK2*, and *ASNS*. (**B**) Representative immunoblots and densitometric quantification of BiP, phosphorylated eIF2α (p-eIF2α), total eIF2α, ATF4, and CHOP. (**C**) Quantitative RT-PCR analysis of *DUSP5* and *4EBP1*. (**D**) Representative immunoblots and densitometric quantification of PI3K/AKT/mTOR-, Raptor-, and ERK1/2-associated proteins. In B and D, phosphoprotein signals were normalized to the corresponding total target protein; BiP, ATF4, and CHOP signals were normalized to β-actin. (**E**) Intracellular reactive oxygen species (ROS) levels measured by DCF-DA staining and flow cytometry, expressed relative to the vehicle-control group. (**F**) Mitochondrial membrane potential assessed by JC-1 staining. Red and green indicate JC-1 aggregates and monomers, respectively; the shift toward green fluorescence indicates mitochondrial membrane depolarization. Carbonyl cyanide *m*-chlorophenyl hydrazone (CCCP) was used as a positive control for mitochondrial depolarization. Data are mean ± standard error of the mean (SEM) from three independent experiments. Technical triplicates for qRT-PCR, ROS, and JC-1 assays were averaged within each independent experiment. Comparisons used one-way ANOVA followed by Tukey’s multiple-comparisons test. * *p* < 0.05, ** *p* < 0.01, *** *p* < 0.001 versus vehicle control.

Based on the transcriptomic findings, representative genes associated with ER stress and the integrated stress response were examined in PSY-treated HCT116 cells. PSY significantly increased, at one or both tested concentrations, the mRNA expression of *ATF4*, *DDIT3*/*CHOP*, *SESN2*, *TRIB3*, *DDIT4*, *STC2*, *CHAC1*, *CARS1*, *PCK2*, and *ASNS* (Figure 3A). Corresponding RNA-seq statistics for the genes assessed by qRT-PCR are provided in Supplementary Table S1B. These transcriptional changes were consistent with the activation of a broader cellular stress-response program.

Immunoblot analysis showed increased BiP, phosphorylated eIF2α (p-eIF2α), ATF4, and CHOP levels following PSY treatment (Figure 3B), consistent with engagement of stress signaling involving eIF2α, ATF4, and CHOP. PSY also increased DUSP5 and *4EBP1* mRNA expression (Figure 3C). Densitometric analysis showed significant reductions in the p-PI3K/PI3K, p-AKT/AKT, p-mTOR/mTOR, and p-Raptor/Raptor ratios at 100 μg/mL PSY. The p-ERK1/2/ERK1/2 ratio was significantly reduced at both 50 and 100 μg/mL (Figure 3D). These results indicate changes in stress-associated and survival-signaling proteins but do not establish the functional contribution of each response to growth inhibition.

Intracellular reactive oxygen species (ROS) levels and mitochondrial membrane potential were assessed in PSY-treated HCT116 cells. PSY increased intracellular ROS levels, as assessed by DCF-DA staining and flow cytometry (Figure 3E), and increased the proportion of cells with mitochondrial membrane depolarization, as assessed by JC-1 staining (Figure 3F). Carbonyl cyanide *m*-chlorophenyl hydrazone (CCCP) was used as a positive control for mitochondrial depolarization (Figure 3F).

Together, these findings show that PSY treatment was accompanied by increased stress-associated markers, intracellular ROS levels, and mitochondrial membrane depolarization, as well as changes in the phosphorylation of PI3K/AKT/mTOR- and ERK-associated proteins in HCT116 cells.

### 2.4. PSY Suppresses HCT116 Xenograft Tumor Growth Without Significant Body-Weight Loss

**Figure 4:**
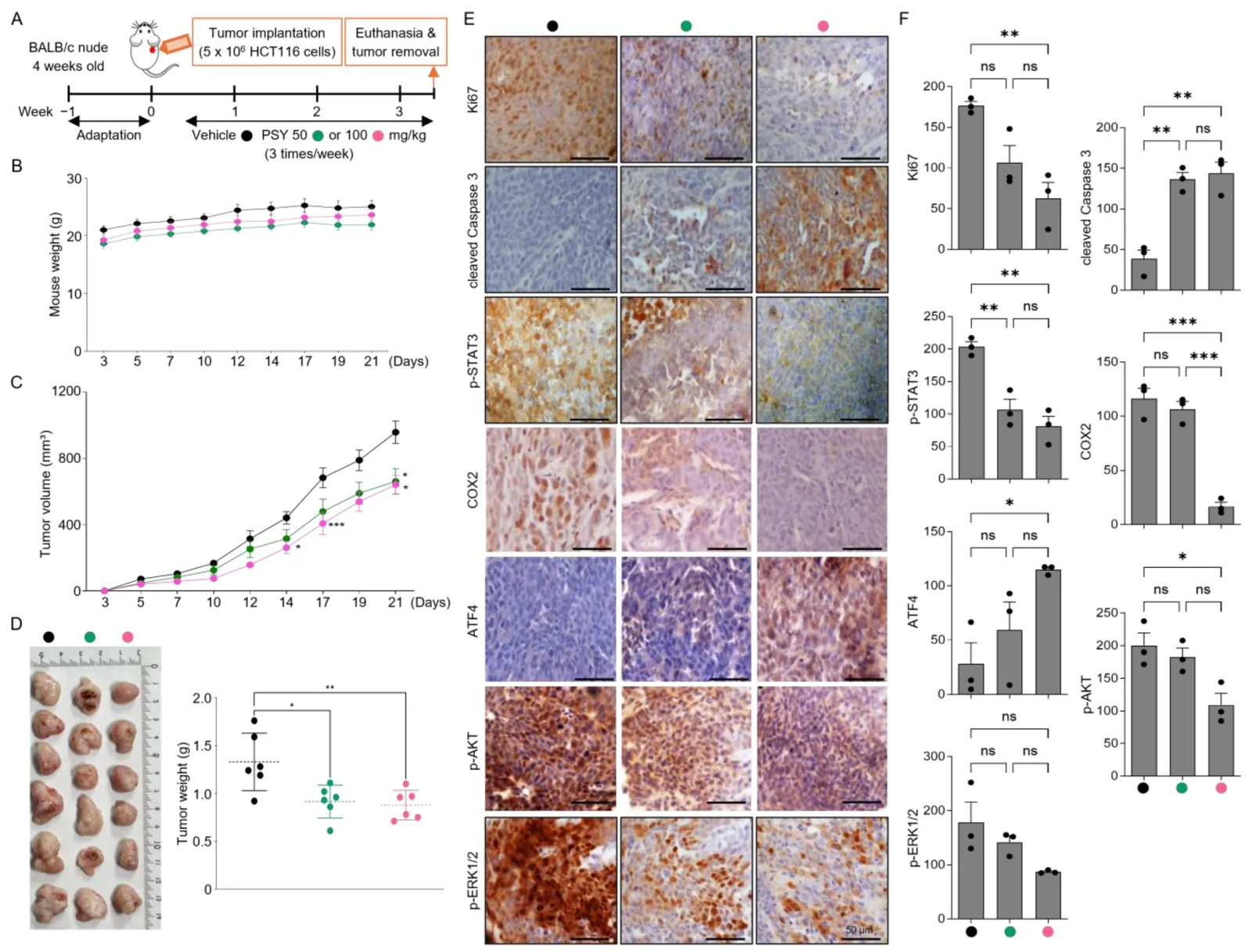
PSY suppresses HCT116 xenograft growth without significant body-weight loss. (**A**) Experimental schedule. Mice bearing HCT116 xenografts received vehicle or PSY (50 or 100 mg/kg) by oral gavage three times weekly for 3 weeks. (**B**) Body weight during the treatment period. (**C**) Tumor-volume progression. (**D**) Excised tumors and final tumor weights. (**E**) Representative tumor sections stained for Ki-67, cleaved caspase-3, p-STAT3, COX2, ATF4, p-AKT, and p-ERK1/2. Scale bar = 50 μm. (**F**) Quantitative IHC analysis using a pixel-based H-score. Two to three fields per mouse were averaged to obtain one value per mouse. Data are mean ± SEM, with *n* = 6 mice per group for (**B**–**D**) and *n* = 3 per group for (**F**). Body-weight and tumor-volume data were analyzed by two-way repeated-measures ANOVA followed by Tukey’s multiple-comparisons test. Final tumor weights and IHC H-scores were analyzed by one-way ANOVA followed by Tukey’s multiple-comparisons test; each IHC marker was analyzed separately. Asterisks above individual bars or points indicate comparisons with vehicle controls; brackets indicate the specified pairwise comparisons. Black, vehicle control; green, PSY 50 mg/kg; and pink, PSY 100 mg/kg. * *p* < 0.05, ** *p* < 0.01, *** *p* < 0.001; ns, not significant.

To evaluate the in vivo antitumor activity of PSY, an HCT116 xenograft model was established in BALB/c nude mice. After subcutaneous inoculation of HCT116 cells and the development of palpable tumors, mice were allocated to receive vehicle or PSY at 50 or 100 mg/kg by oral gavage three times per week for 3 weeks (Figure 4A).

Body weights remained comparable among the treatment groups throughout the experimental period, with no significant differences observed between the vehicle-control and PSY-treated groups (Figure 4B). No treatment-related adverse events were observed, and no animal reached the predefined humane endpoints during the study. In contrast, tumor-volume progression was significantly reduced in the PSY-treated groups compared to the vehicle-control group (Figure 4C). Images of excised tumors showed a reduction in tumor burden, and final tumor weights were significantly lower in the PSY-treated groups than in the vehicle-control group (Figure 4D). These findings indicate that orally administered PSY suppresses HCT116 xenograft tumor growth under the experimental conditions used.

Representative immunohistochemical images and quantitative H-score analysis are presented in Figure 4E,F. PSY-treated tumors showed lower p-STAT3 H-scores and higher cleaved caspase-3 H-scores than vehicle controls at both 50 and 100 mg/kg. At 100 mg/kg, p-AKT, Ki-67, and COX2 H-scores were also lower, and ATF4 H-scores were higher, than those in vehicle controls. These comparisons were significant after Tukey adjustment (*p* < 0.05). COX2 H-scores were lower at 100 than at 50 mg/kg. Mean p-ERK1/2 H-scores were numerically lower in the PSY-treated groups, but the differences from vehicle controls were not statistically significant (adjusted *p* = 0.5163 and 0.0672 for 50 and 100 mg/kg, respectively). These findings associate PSY treatment with changes in proliferation-, apoptosis-, and stress-associated markers in xenograft tumors.

### 2.5. PSY Alters Lipid-Related Transcripts and Proteins and the Abundance of Selected Tumor Fatty Acids

**Figure 5:**
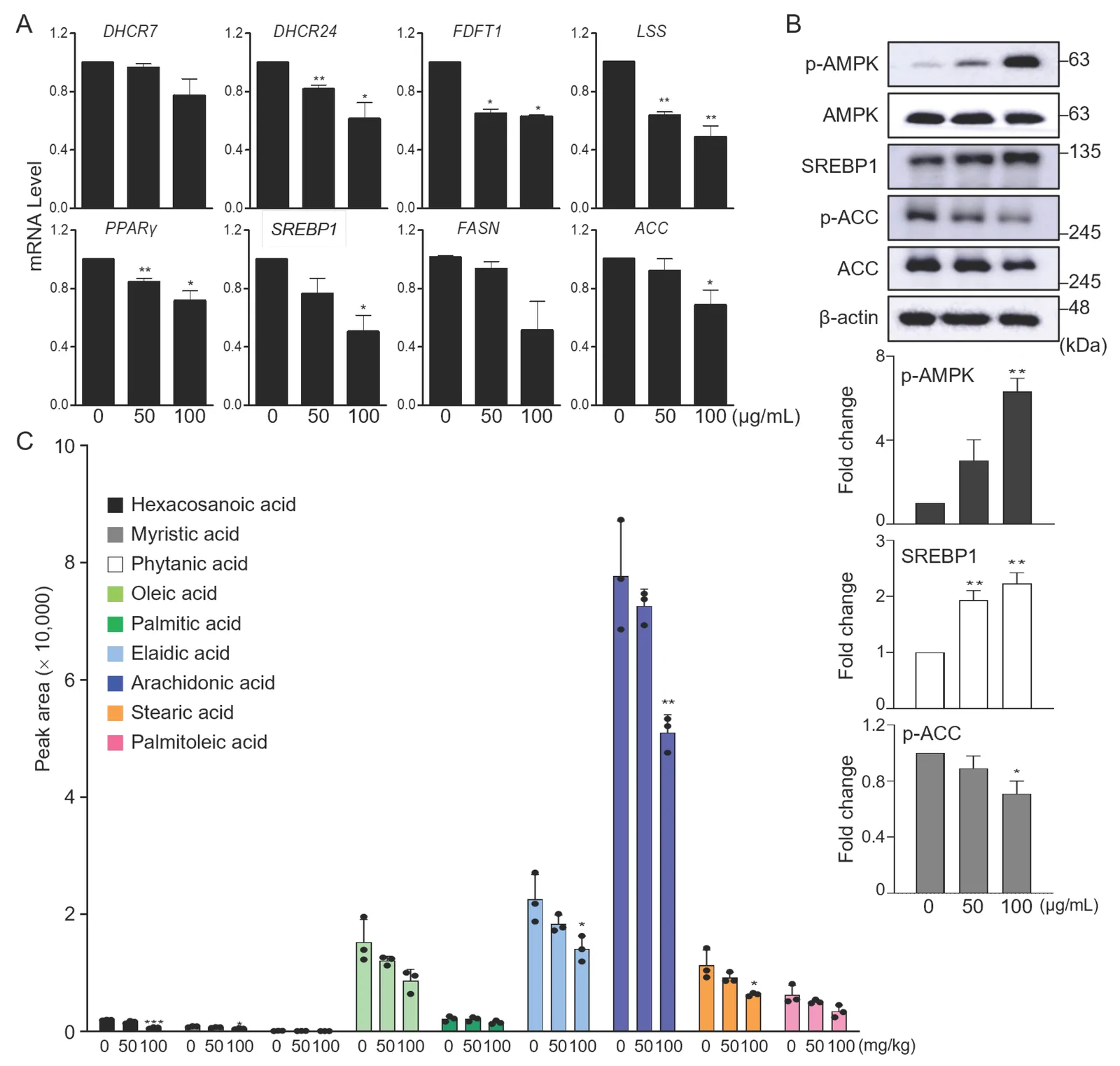
PSY alters lipid-related transcripts and proteins and the abundance of selected tumor fatty acids. (**A**) Quantitative RT-PCR analysis of *DHCR7*, *DHCR24*, *FDFT1*, *LSS*, *PPARG*, *SREBP1*, *FASN*, and *ACC* in HCT116 cells treated with PSY at the indicated concentrations. Data are from three independent experiments, each analyzed in technical triplicate. (**B**) Representative immunoblots and densitometric quantification from three independent experiments. Phosphorylated AMPK (Thr172) and ACC (Ser79) were normalized to their corresponding total proteins; the approximately 135 kDa SREBP1 band was normalized to β-actin. (**C**) LC–MS/MS fatty-acid profiles of three independent xenograft tumors per group, expressed as integrated peak areas for relative comparisons. The fatty-acid groups are shown from left to right in the following order: hexacosanoic acid, myristic acid, phytanic acid, oleic acid, palmitic acid, elaidic acid, arachidonic acid, stearic acid, and palmitoleic acid. Within each group, 0, 50, and 100 indicate vehicle control, PSY 50 mg/kg, and PSY 100 mg/kg, respectively. Data are mean ± SEM. Comparisons used one-way ANOVA followed by Tukey’s multiple-comparisons test. * *p* < 0.05, ** *p* < 0.01, *** *p* < 0.001 versus vehicle control.

Because exploratory transcriptomic analysis highlighted lipid-related processes, qRT-PCR was used to examine genes involved in cholesterol and fatty-acid biosynthesis. *DHCR24*, *FDFT1*, *LSS*, and *PPARG* mRNA levels were significantly reduced at both 50 and 100 μg/mL PSY, whereas significant reductions in *SREBP1* and *ACC* mRNA levels were observed at 100 μg/mL. *DHCR7* and *FASN* mRNA levels did not differ significantly from vehicle controls at either concentration (Figure 5A). In the complementary RNA-seq analysis, *DHCR7*, *DHCR24*, *FDFT1*, *LSS*, and *SREBP1* met adjusted *p* < 0.05, whereas *FASN*, *ACC*, and *PPARG* did not (Supplementary Table S1B).

Densitometric analysis showed a significant increase in the p-AMPK (Thr172)/AMPK ratio and a significant decrease in the p-ACC (Ser79)/ACC ratio at 100 μg/mL PSY, while total ACC abundance was maintained. The approximately 135 kDa SREBP1 band, normalized to β-actin, was significantly increased at both 50 and 100 μg/mL (Figure 5B). Thus, AMPK and ACC phosphorylation changed in opposite directions, and the increase in the detected SREBP1 protein band contrasted with the reduction in *SREBP1* mRNA at 100 μg/mL.

To examine tumor fatty-acid abundances in vivo, LC-MS/MS analysis was performed using xenograft tissues. Tumors from mice receiving 100 mg/kg PSY showed significantly lower relative abundances of hexacosanoic acid, myristic acid, elaidic acid, arachidonic acid, and stearic acid than vehicle controls (Figure 5C). The other measured fatty acids showed smaller or less consistent changes among the treatment groups.

Together, these findings show changes in lipid-related transcripts and proteins in HCT116 cells and the relative abundance of selected fatty acids in xenograft tumors.

### 2.6. PSY Shows the Most Consistent CI-Defined In Vitro Synergistic Interaction with 5-FU Among the Tested Chemotherapeutic Agents

**Figure 6:**
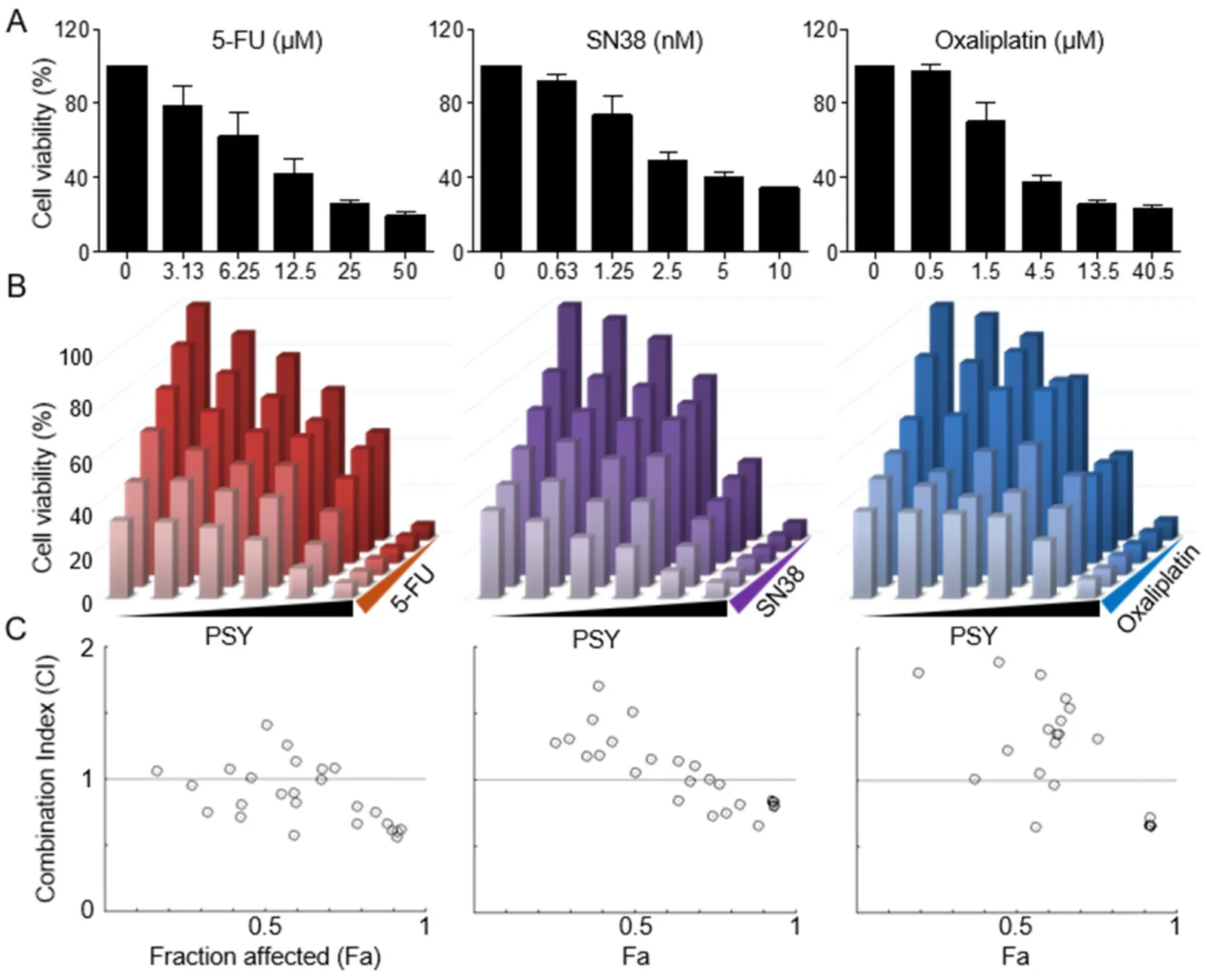
Drug-combination analysis of PSY with chemotherapeutic agents in HCT116 cells. (**A**) Cell viability after 72 h of treatment with the indicated concentrations of 5-fluorouracil (5-FU), SN38, or oxaliplatin. (**B**) Cell viability after 72 h of combined treatment with PSY and each chemotherapeutic agent at the concentration pairs listed in Table 1. (**C**) Combination index (CI) plotted against fraction affected (Fa). CI < 1, CI = 1, and CI > 1 indicate synergistic, additive, and antagonistic interactions, respectively. Cell viability assays and combination experiments were performed independently three times, with triplicate wells per condition in each experiment. Technical-well values were averaged within each experiment, and viability is presented as mean ± SEM in (**A**) and as mean values in (**B**). CI values are point estimates calculated from the mean viability across these experiments; they are not means of three independently calculated CI values.

To evaluate the effects of combining PSY with standard CRC chemotherapeutic agents, HCT116 cells were treated with 5-FU, SN38, or oxaliplatin alone or in combination with PSY. Each chemotherapeutic agent reduced cell viability in a concentration-dependent manner (Figure 6A).

Combination-index (CI) analysis indicated agent- and concentration-dependent interactions between PSY and the three chemotherapeutic agents (Figure 6B,C and Table 1). CI values below 1 were observed for 17 of 25 tested PSY–5-FU pairs, 12 of 25 PSY–SN38 pairs, and 6 of 25 PSY–oxaliplatin pairs. These counts describe the tested matrices and do not constitute a statistical comparison between drugs. On this basis, PSY–5-FU was selected for further cellular and molecular characterization.

PSY at 25 μg/mL combined with 5-FU at 5 μM was used in subsequent experiments because this combination yielded a CI-defined in vitro synergistic interaction (Fa = 0.503; CI = 0.577) under the tested conditions at relatively low concentrations while maintaining a suitable dynamic range for downstream analyses.

**Figure 7:**
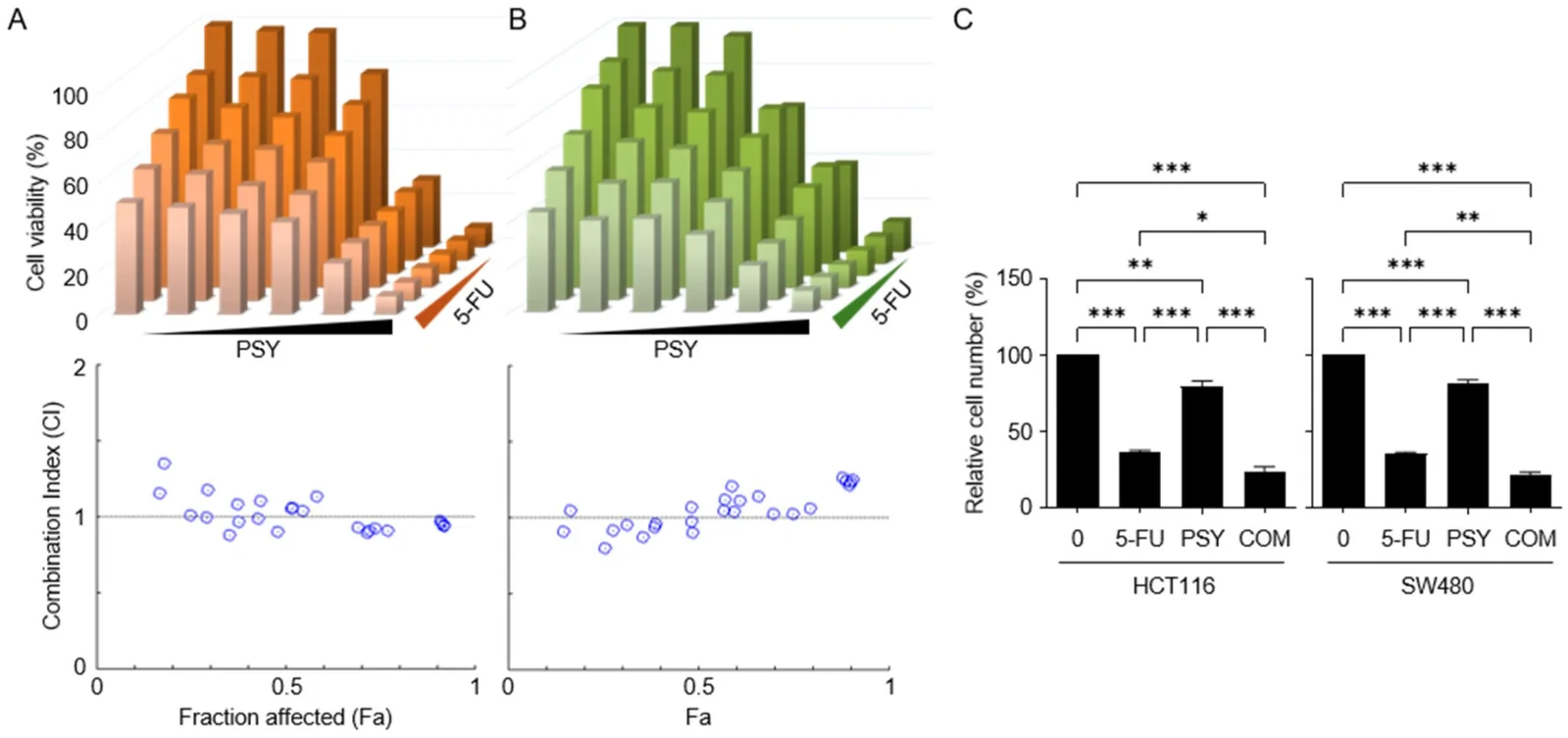
PSY–5-FU combination responses in colorectal cancer cells assessed by MTT and direct cell counting. (**A**,**B**) MTT-based cell viability and CI analyses in HT-29 (**A**) and SW480 (**B**) cells after 72 h of treatment. PSY concentrations were 0, 12.5, 25, 50, 100, and 200 μg/mL. The corresponding 5-FU concentrations were 0, 2.1875, 4.375, 8.75, 17.5, and 35 μM for HT-29 cells and 0, 4.375, 8.75, 17.5, 35, and 70 μM for SW480 cells. Upper plots show mean cell viability expressed as a percentage of the vehicle control. Lower plots show CI values against Fa. CI values were calculated from the mean viability across three independent experiments and represent point estimates. The horizontal line indicates CI = 1. MTT experiments were performed independently three times, with triplicate wells per condition in each experiment. Technical replicate values were averaged within each experiment. (**C**) Direct viable cell counting by trypan blue exclusion after 72 h in HCT116 cells treated with PSY (25 μg/mL), 5-FU (5 μM), or their combination and in SW480 cells treated with PSY (12.5 μg/mL), 5-FU (8.75 μM), or their combination. Cell counts were normalized to the corresponding vehicle control within each independent experiment. Data are presented as mean ± SEM from three independent experiments, with one well per condition in each experiment. Statistical comparisons were performed separately for each cell line using one-way ANOVA followed by Tukey’s multiple comparisons test. Brackets indicate the groups compared. * *p* < 0.05, ** *p* < 0.01, and *** *p* < 0.001. 0, vehicle control; COM, PSY plus 5-FU.

To extend the combination analysis to additional CRC cell lines, PSY–5-FU concentration matrices were evaluated in HT-29 and SW480 cells. MTT-based CI analysis revealed concentration-dependent interaction patterns in both cell lines (Figure 7A,B). CI values below 1 were observed for 15 of 25 tested concentration pairs in HT-29 cells and 9 of 25 pairs in SW480 cells. CI values ranged from 0.881 to 1.354 in HT-29 cells and from 0.801 to 1.263 in SW480 cells, indicating that the interaction varied across the tested concentration matrices.

Direct cell counting provided a complementary assessment of treatment-associated changes in cell number. At the selected concentrations, combined treatment resulted in significantly lower cell counts than either single treatment in both HCT116 and SW480 cells (Figure 7C). Mean relative cell counts in HCT116 cells were 35.8%, 79.4%, and 23.4% of vehicle control following 5-FU, PSY, and combined treatment, respectively. The corresponding values in SW480 cells were 35.1%, 81.0%, and 21.5%.

Cell-free MTT controls showed low absorbance, with no statistically significant differences among treatment conditions under the conditions tested (Supplementary Figure S1).

### 2.7. The PSY–5-FU Combination Enhances Apoptosis and Alters Stress-Associated and Survival-Signaling Markers in HCT116 Cells

**Figure 8:**
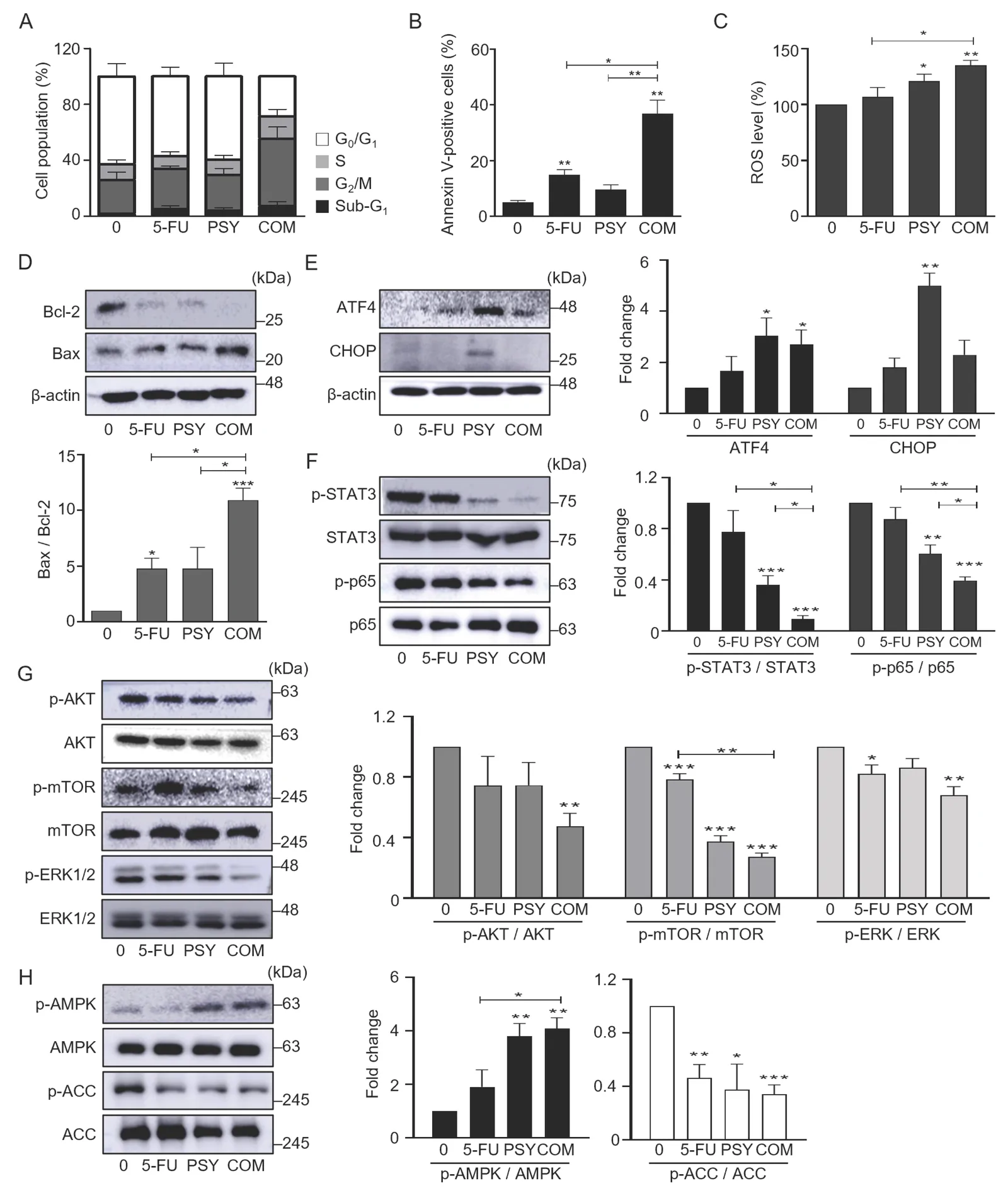
The PSY–5-FU combination enhances apoptosis and alters stress-associated and survival-signaling markers in HCT116 cells. Cells were treated with vehicle, PSY (25 μg/mL), 5-FU (5 μM), or the combination of PSY and 5-FU. (**A**) Cell-cycle distribution after 48 h of treatment. (**B**) Annexin V/PI analysis of apoptotic cells after 48 h of treatment. (**C**) Intracellular ROS levels measured by DCF-DA staining after 24 h of treatment. (**D**) Representative immunoblots and densitometric quantification of Bax and Bcl-2, with results expressed as the Bax/Bcl-2 ratio. (**E**) Representative immunoblots and quantification of the ER stress-related proteins ATF4 and CHOP. (**F**) Representative immunoblots and quantification of phosphorylated and total STAT3 and NF-κB p65. (**G**) Representative immunoblots and quantification of phosphorylated and total AKT, mTOR, and ERK1/2. (**H**) Representative immunoblots and quantification of phosphorylated and total AMPK and ACC. Immunoblots and densitometry in (**D**–**H**) are from three independent experiments. Phosphoproteins were normalized to the corresponding total proteins, and other proteins were normalized to β-actin; (**D**) shows the Bax/Bcl-2 ratio. Other assays were performed independently at least three times. Data are mean ± SEM and were analyzed by one-way ANOVA followed by Tukey’s multiple-comparisons test. Asterisks above individual bars indicate comparisons with the vehicle-control group, whereas asterisks above brackets indicate the specific pairwise comparisons. Only statistically significant comparisons are annotated; comparisons without asterisks were not statistically significant. * *p* < 0.05, ** *p* < 0.01, and *** *p* < 0.001.

To characterize the cellular responses associated with the CI-defined in vitro synergistic interaction between PSY and 5-FU, HCT116 cells were treated with vehicle, PSY, 5-FU, or their combination. Cell-cycle analysis showed a higher sub-G1 fraction in the combination group (Figure 8A). Consistently, Annexin V/PI staining demonstrated that the combination increased the proportion of apoptotic cells compared with the corresponding single-agent treatments (Figure 8B). These findings support an enhanced apoptotic response to the PSY–5-FU combination.

Intracellular ROS levels, as assessed by DCF-DA staining and flow cytometry, were significantly higher in the combination group than in the 5-FU-alone group, but did not differ significantly from those in the PSY-alone group (Figure 8C). The Bax/Bcl-2 ratio was significantly higher in the combination group than in either single-treatment group (Figure 8D). ATF4 and CHOP levels did not differ significantly between the combination and either single treatment (Figure 8E). Thus, the enhanced apoptotic response was not accompanied by a statistically significant additional increase in these two stress-associated proteins.

Immunoblot analysis further showed that the combination reduced the p-STAT3/STAT3 and p-NF-κB p65/total NF-κB p65 ratios more strongly than either single treatment (Figure 8F), indicating greater reductions in these phosphorylation markers at the tested concentrations.

For p-AKT/AKT, only the combination group showed a significant reduction relative to the control group, and the combination did not differ significantly from either single treatment (Figure 8G). The p-mTOR/mTOR ratio was significantly lower in the combination group than with 5-FU alone, but not with PSY alone. For p-ERK/ERK and p-ACC/ACC, significant differences were confined to comparisons with the control group, with no significant differences between the combination and either single treatment (Figure 8G,H). The p-AMPK/AMPK ratio was significantly higher with the combination than with 5-FU alone, whereas the difference from PSY alone was not significant (Figure 8H).

Together, the combination-index analysis identified a CI-defined in vitro synergistic interaction between PSY and 5-FU under the tested conditions, while the cellular analyses characterized responses associated with the combination. The combination was accompanied by enhanced apoptosis, increased intracellular ROS levels, and changes in inflammatory, survival, and metabolic signaling markers. These observations do not establish which molecular changes are required for the interaction.

## 3. Discussion

The present study extends our previous STAT3-focused characterization of PSY by examining additional molecular and metabolic responses, antitumor activity in an HCT116 xenograft model, and interactions with chemotherapy in vitro. PSY treatment was associated with changes in stress-related proteins, survival-signaling markers, lipid-biosynthetic transcripts, and selected tumor fatty acids. Oral PSY reduced xenograft growth, and combination-index analysis identified CI-defined in vitro synergistic interactions with 5-FU at multiple concentration pairs under the tested conditions. These findings broaden the description of PSY activity, while leaving the causal relationships among the molecular responses and their contributions to growth inhibition unresolved.

Exploratory transcriptomic profiling highlighted stress-associated and metabolic processes that guided subsequent experiments. The original selection of 140 candidates used nominal *p*-value, fold-change, and expression criteria; none met BH-adjusted *p* < 0.05 in the original *t*-test analysis. A complementary DESeq2 analysis of the same samples identified 262 genes at adjusted *p* < 0.05, including 108 of the original candidates, with concordant directions of change for all 140. This agreement supports consistency across analytical approaches but does not constitute independent experimental replication. Several selected responses were also observed by qRT-PCR and immunoblotting, although concordance was not universal. With only two biological replicates per group, the RNA-seq findings should remain hypothesis-generating.

ER stress and the integrated stress response emerged as prominent components of the cellular response to PSY. PSY increased the expression of multiple stress-response genes, including *ATF4*, *DDIT3*/*CHOP*, *DDIT4*, *TRIB3*, *SESN2*, *CHAC1*, *ASNS*, *PCK2*, and *CARS1*. Increased levels of BiP, p-eIF2α, ATF4, and CHOP protein were also consistent with engagement of stress signaling involving eIF2α, ATF4, and CHOP. ER stress and the UPR exert context-dependent effects in cancer: moderate activation can facilitate adaptation and survival, whereas excessive or prolonged stress may contribute to cell death [6,7,18]. In the present study, these stress-associated responses were accompanied by increased intracellular ROS levels and mitochondrial membrane depolarization. ROS-associated ER-stress responses have also been reported in HCT116 cells treated with other anticancer agents; for example, MHY446 induced ROS generation, ER-stress signaling, and apoptosis, with ROS-scavenging experiments supporting functional coupling between these responses [19]. However, these concurrent changes do not establish their causal ordering or demonstrate that ER stress or the eIF2α–ATF4–CHOP response is required for PSY-associated growth inhibition or apoptosis. Targeted pharmacological inhibition, genetic perturbation, or rescue experiments would be needed to determine the functional contributions and relationships of these responses.

PSY treatment also reduced phosphorylation-associated signals for PI3K, AKT, mTOR, and ERK1/2. These pathways participate in cancer-cell survival and responses to metabolic, oxidative, and proteotoxic stress [2,5,20]. Increased *DUSP5* and *4EBP1* mRNA expression provided additional evidence of changes in signaling-related gene expression, although transcript levels alone do not establish the activity of their encoded proteins. AMPK-dependent phosphorylation of Raptor at Ser722 and Ser792 contributes to mTORC1 inhibition during energy stress [21]. Accordingly, the reduced p-Raptor (Ser792) signal observed following PSY treatment should not be interpreted as a marker of mTORC1 inhibition. The functional significance of this change in the response to PSY remains to be established. Overall, the results show concurrent changes in stress-associated and survival-signaling markers. They do not establish whether these changes mediate growth inhibition, arise as secondary responses, or alter the capacity of cells to compensate for PSY-induced stress.

Oral administration of PSY reduced HCT116 xenograft growth without significant body-weight loss under the experimental conditions used. Quantitative IHC showed lower p-STAT3 and higher cleaved caspase-3 H-scores at both doses. At 100 mg/kg, lower p-AKT, Ki-67, and COX2 and higher ATF4 H-scores were also observed relative to vehicle controls. Although mean p-ERK1/2 H-scores were lower in the PSY-treated groups, these differences were not statistically significant. The IHC findings associate PSY treatment with changes in proliferation-, apoptosis-, and stress-related markers in tumor tissue, but do not establish that an individual signaling response mediates tumor-growth inhibition. Histological analysis used three independent tumors per group, and the absence of significant body-weight loss should not be interpreted as a comprehensive assessment of systemic safety.

The lipid-related measurements showed selective and partly discordant responses to PSY. Altered lipogenic signaling has functional relevance to CRC biology; for example, PTPRO loss has been reported to promote CRC growth and metastasis through activation of an AKT/mTOR/SREBP1/ACC1-associated lipogenic program [22]. qRT-PCR identified reductions in *DHCR24*, *FDFT1*, *LSS*, *PPARG*, *SREBP1*, and *ACC* at one or both concentrations, whereas *DHCR7* and *FASN* changes were not statistically significant. At 100 μg/mL, the p-AMPK (Thr172)/AMPK ratio increased while the p-ACC (Ser79)/ACC ratio decreased, with total ACC abundance maintained. Because Ser79 is an inhibitory phosphorylation site on ACC [23], this decrease does not support ACC inhibition through the canonical AMPK–ACC mechanism. Reduced inhibitory phosphorylation could favor ACC activity, but neither ACC enzyme activity nor lipogenic flux was measured. ACC phosphorylation reflects the balance of kinase and phosphatase activities; protein phosphatase 4, for example, can dephosphorylate ACC at Ser79 in hepatic models [24]. Such regulation is a possible explanation for the divergent phosphorylation responses, but was not examined here. Together with reduced Raptor Ser792 phosphorylation, these findings caution against inferring uniform activation of downstream AMPK responses from increased AMPK Thr172 phosphorylation alone.

The increase in the approximately 135 kDa SREBP1 band also requires distinction between protein abundance and transcriptional activity. Its apparent molecular mass is consistent with a precursor-sized species rather than the smaller cleaved nuclear form [25]; the present whole-cell immunoblots do not establish increased mature nuclear SREBP1. AMPK has been shown to inhibit SREBP1 processing through direct phosphorylation and through stabilization of insulin-induced gene proteins in other experimental systems [25,26]. Precursor accumulation with limited processing is therefore one possible explanation for increased SREBP1 immunoreactivity despite reduced *SREBP1* mRNA and selected lipid-related transcripts. Differences in translation or protein turnover could also contribute to the mRNA–protein discordance for SREBP1 and ACC. These possibilities remain untested because precursor-to-mature processing, nuclear localization, protein turnover, and lipogenic flux were not assessed. The data support changes in lipid-related regulation but do not establish a linear AMPK–ACC–SREBP1 mechanism or demonstrate that reduced lipid synthesis mediates growth inhibition.

Fatty-acid profiling showed lower relative abundance of selected fatty acids in xenograft tumors from mice receiving 100 mg/kg PSY, including hexacosanoic acid, myristic acid, elaidic acid, arachidonic acid, and stearic acid. The arachidonic acid finding is of interest in the context of lipid-mediated inflammation and COX2-related CRC biology [3,4,8,27]. However, profiling of three tumors per group provides a limited assessment of tumor lipid composition and does not measure lipid synthesis or turnover directly. The observed differences could reflect changes in tumor growth, cellular composition, or metabolic state. Whether these lipid changes contribute to the antitumor effect, or occur as a consequence of it, remains unresolved.

The combination experiments identified a CI-defined in vitro synergistic interaction between PSY and 5-FU at multiple tested concentration pairs in HCT116 cells. At the selected concentrations, the combination increased Annexin V-positive apoptosis and the Bax/Bcl-2 ratio relative to the single treatments and was accompanied by higher intracellular ROS levels than those observed with 5-FU alone; the difference from PSY alone was not significant. ATF4 and CHOP did not differ significantly between the combination and either single treatment. The combination also reduced STAT3 and NF-κB p65 phosphorylation relative to the single treatments, whereas comparisons involving other signaling markers were less uniform. These results describe molecular responses accompanying the combination but do not establish which responses are required for the interaction. Because the agents were administered together, the experiments also do not establish a directional sensitization mechanism. Comparable context dependence has been reported for other natural compound–5-FU combinations in CRC. For example, diosmetin combined with 5-FU showed a CI-defined synergistic interaction in HCT116 cells but an approximately additive interaction in HT-29 cells, together with enhanced apoptotic responses [28]. Tangeretin has likewise been reported to enhance the antitumor response to 5-FU in CRC models, with accompanying modulation of the miR-21/PTEN/PI3K-AKT axis and autophagy [29]. These studies illustrate that natural product–5-FU interactions can involve distinct cellular responses, but they do not imply that the same mechanisms underlie the PSY–5-FU interaction observed here.

The additional experiments extended the assessment of PSY–5-FU interactions to HT-29 and SW480 cells. CI values varied with concentration, and several estimates were close to 1, indicating that CI-defined synergistic interactions were concentration- and cell-line-dependent rather than uniform across the three CRC cell lines. Direct cell counting showed significantly lower cell numbers with combined treatment than with either agent alone in HCT116 and SW480 cells, providing evidence complementary to the MTT measurements. Cell-free MTT controls also showed low absorbance without significant treatment-related differences under the conditions tested. These findings strengthen the experimental basis for evaluating the combination, while the molecular responses characterized in HCT116 cells remain to be examined in the additional cell lines.

**Figure 9:**
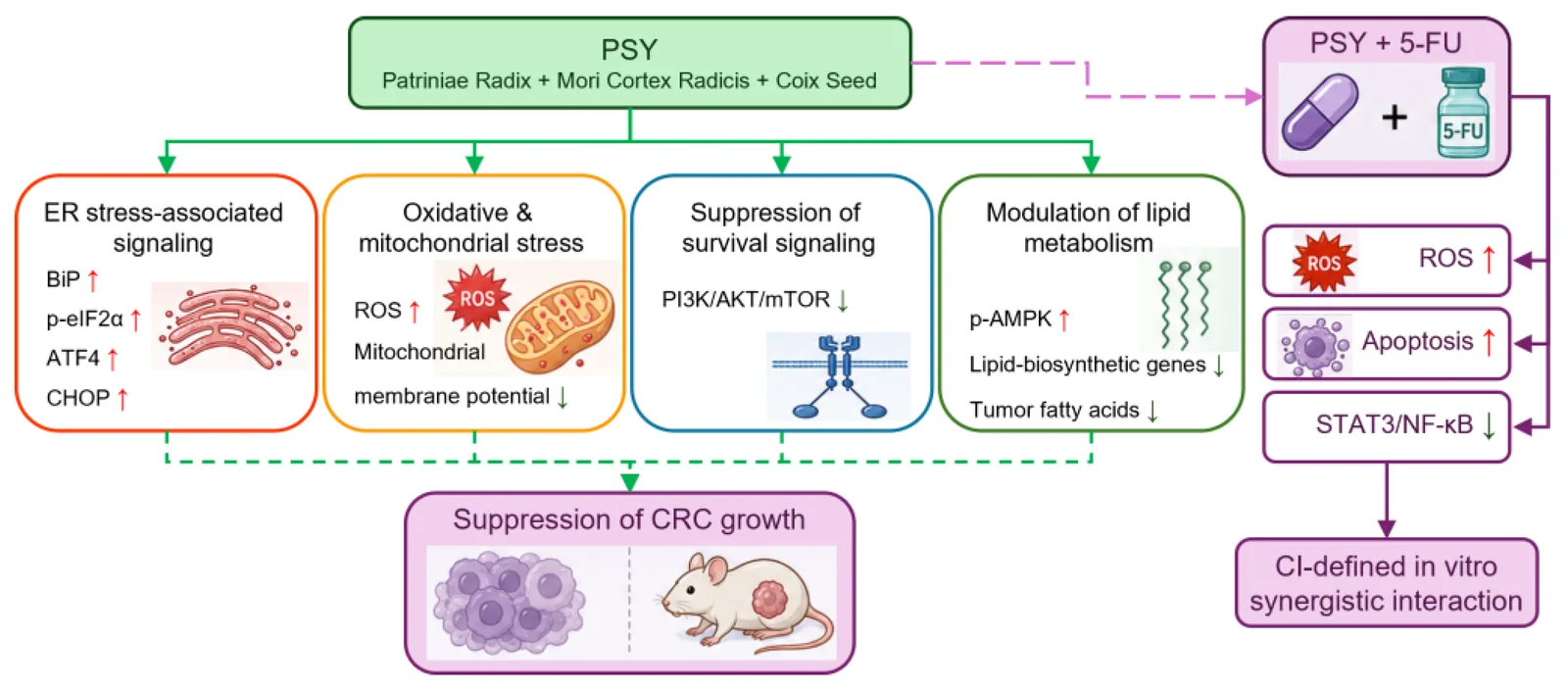
Summary of responses observed following PSY treatment and PSY–5-FU combination treatment. PSY was associated with changes in stress-related proteins, intracellular ROS levels, mitochondrial membrane potential, survival-signaling markers, and lipid-related transcripts and proteins. PSY alone reduced HCT116 xenograft growth and altered selected IHC markers and fatty-acid abundances. In vitro combination experiments identified CI-defined synergistic interactions with 5-FU under the tested conditions and accompanying changes in apoptosis and signaling markers. Upward and downward arrows indicate increases and decreases, respectively, in the measured markers or responses. Solid connector arrows indicate treatment-associated responses within the depicted experimental setting, whereas dashed connector lines indicate schematic links between findings obtained in distinct experimental settings and are not intended to imply direct causality or pathway dependence. Some illustrative graphical elements in this figure (e.g., cells, mouse, endoplasmic reticulum, mitochondrion, etc.) were generated using ChatGPT Images (OpenAI; https://chatgpt.com/; version available in July 2026; accessed between 15 and 20 July 2026). The overall layout, text, arrows, and scientific organization were created and assembled by the authors.

CRC biology involves interactions among inflammatory signaling, stress adaptation, survival pathways, and metabolic regulation [2,3,5,7,9,30]. The present study extends our previous characterization of PSY [14] by documenting a broader set of treatment-associated responses, changes in selected tumor fatty acids, xenograft growth inhibition, and an in vitro interaction with 5-FU. Figure 9 summarizes these observations across experimental systems. The study does not identify the direct molecular targets of the extract or establish a causal sequence connecting the measured pathways to the growth response. Its main contribution is therefore the expanded experimental characterization of PSY and the identification of questions for subsequent functional and combination studies.

Several limitations should be considered. Although PSY–5-FU interactions were evaluated in HCT116, HT-29, and SW480 cells, molecular characterization was centered on HCT116 cells, and the combination was evaluated only in vitro. RNA sequencing included two biological replicates per group, and the complementary reanalysis does not remove this limitation. The xenograft study used a single HCT116 model in immunodeficient male mice, without formal randomization or blinding; tissue-level IHC and fatty-acid profiling each used three tumors per group. All biological experiments used one PSY extract batch, and marker concentrations and between-batch reproducibility were not assessed. Functional perturbation or rescue experiments are needed to establish pathway contributions, while studies in additional CRC models, in vivo combination experiments, and more extensive safety and exposure assessments are needed to define the broader applicability of the findings.

## 4. Materials and Methods

### 4.1. Cell Culture

The human CRC cell lines HCT116 (KCLB No. 10247; RRID: CVCL_0291), HT-29 (KCLB No. 30038; RRID: CVCL_0320), and SW480 (KCLB No. 10228; RRID: CVCL_0546) were obtained from the Korean Cell Line Bank (Seoul, Republic of Korea) and cultured in RPMI-1640 medium supplemented with 10% fetal bovine serum and 1% antibiotic–antimycotic solution, as previously described [14]. Cells were maintained at 37 °C in a humidified atmosphere containing 5% CO_2_. Cell numbers and viability were determined by trypan blue exclusion using either a manual hemocytometer or an LUNA-FL automated cell counter (Logos Biosystems, Gunpo, Gyeonggi-do, Republic of Korea).

### 4.2. Preparation and Chemical Marker Verification of PSY

Dried Patriniae Radix (root of *Patrinia scabiosaefolia* Link), Mori Cortex Radicis (root bark of *Morus alba* L.), and Coix Seed (seed of *Coix lacryma-jobi* var. *ma-yuen* (Rom.Caill.) Stapf) were purchased from Omniherb (Daegu, Republic of Korea). Voucher specimens of the three medicinal materials were deposited in the herbarium of the Korean Medicine Clinical Trial Center, Kyung Hee University Korean Medicine Hospital (Seoul, Republic of Korea).

PSY was prepared as previously described [14]. Briefly, Patriniae Radix, Mori Cortex Radicis, and Coix Seed were combined at a weight ratio of 5:3:5 (37.5, 22.5, 37.5 g, respectively) and extracted by sonication in 877.5 mL of ethanol at room temperature. The extract was filtered, concentrated under reduced pressure at 40 °C, and lyophilized. The yield of the dried extract was 3.2% relative to the total weight of the starting herbal materials. The lyophilized extract was dissolved in dimethyl sulfoxide (DMSO) to prepare a 500 mg/mL stock solution. All biological experiments in the present study used a single batch of PSY ethanol extract prepared specifically for this study.

Chemical analysis was performed by ultra-performance liquid chromatography coupled with electrospray ionization quadrupole time-of-flight tandem mass spectrometry (UPLC-ESI-QTOF MS/MS) as previously described [14]. Loganic acid (ChemFaces, Wuhan, China), morusin (Biopurity Phytochemicals, Ltd., Chengdu, China), and linoleic acid (Sigma-Aldrich, St. Louis, MO, USA) were qualitatively identified by comparing their retention times and MS/MS fragmentation patterns with those of authentic reference standards, with the corresponding chromatograms and MS/MS spectra presented in Figure 1.

### 4.3. RNA Sequencing and Data Analysis

HCT116 cells were treated with PSY (50 μg/mL) or vehicle (0.1% DMSO) for 24 h. RNA sequencing was performed using two independent biological replicates per treatment group (*n* = 2). Total RNA was extracted using TRIzol reagent (Thermo Fisher Scientific, Waltham, MA, USA), and RNA quality was assessed using an Agilent 2100 Bioanalyzer (Agilent Technologies, Santa Clara, CA, USA). Libraries were prepared using the QuantSeq 3′ mRNA-Seq Library Prep Kit (Lexogen, Greenland, NH, USA), and single-end sequencing was performed on an Illumina NextSeq 500 platform by Ebiogen Inc. (Seoul, Republic of Korea). The sequencing dataset was deposited in National Center for Biotechnology Information (NCBI) Gene Expression Omnibus (GEO) under accession number GSE285901.

Sequencing reads were aligned to the human reference genome (hg19), followed by gene-level read counting. For the original exploratory analysis, normalized expression values generated using TMM and CPM normalization were compared by two-sided, equal-variance Student’s *t*-tests on the linear expression scale. BH adjustment was applied across all 18,032 entries with computable *p* values. Exploratory candidates were selected using a PSY/control expression ratio ≥ 1.5 or ≤2/3, unadjusted *p* < 0.05, and mean normalized log_2_ expression ≥ 4 in at least one group. Heatmaps and volcano plots were generated using ExDEGA (Ebiogen Inc., Seoul, Republic of Korea; https://www.e-biogen.com/) [31].

For complementary analysis, raw counts from individual samples were analyzed using DESeq2 [32] version 1.52.0 with treatment condition (control or PSY) as the sole explanatory variable. Differential expression was evaluated in PSY-treated samples relative to controls. Entries with fewer than 10 total counts across the four samples were excluded, leaving 14,842 entries. Counts were normalized using median-ratio size factors, and two-sided Wald *p* values were adjusted using the BH method with independent filtering; 10,813 entries had adjusted *p* values. Supplementary Table S1A summarizes the 140 exploratory candidates, Supplementary Table S1B provides RNA-seq statistics for genes assessed by qRT-PCR, and Supplementary Table S1C provides the complete gene-level results from both analyses.

Protein–protein interaction (PPI) and functional enrichment analyses were performed using STRING version 11.5 (https://string-db.org). The 140 exploratory candidates were uploaded to STRING, and interactions were filtered using a high-confidence interaction score of ≥0.900. The resulting network was examined to identify clusters of highly interconnected genes. Gene Ontology (GO) biological-process and Kyoto Encyclopedia of Genes and Genomes (KEGG) pathway enrichment analyses were also performed, with a false discovery rate of <0.05 considered statistically significant. Enrichment adjusted *p* values refer to term-level tests and are distinct from the gene-level adjusted *p* values reported in Supplementary Table S1.

### 4.4. Quantitative Real-Time PCR

Total RNA was extracted from cultured cells or xenograft tumor tissues and reverse-transcribed into complementary DNA using the PrimeScript First Strand cDNA Synthesis Kit (Takara Korea Biomedical Inc., Seoul, Korea). Quantitative real-time PCR (qRT-PCR) was performed using qPCRBIO SyGreen Mix (PCR Biosystems Inc., East Hartford, CT, USA) on a StepOnePlus Real-Time PCR System (Applied Biosystems, Waltham, MA, USA).

All qRT-PCR experiments were performed independently at least three times, with each biological sample analyzed in technical triplicate. Relative gene-expression levels were normalized to those of GAPDH and calculated using the 2^−ΔΔCt^ method [33]. Results are presented as fold changes relative to the corresponding vehicle-control group. The primer sequences used in this study are listed in Table 2.

### 4.5. Western Blot Analysis

Whole-cell lysates were prepared and subjected to sodium dodecyl sulfate–polyacrylamide gel electrophoresis (SDS-PAGE) and immunoblotting, as described previously [14]. The following primary antibodies were obtained from Cell Signaling Technology (Danvers, MA, USA): ATF4 (11815S), CHOP (2895S), p-eIF2α (Ser51) (3597S), eIF2α (5324S), BiP (3177S), p-PI3K p85 (Tyr458)/p55 (Tyr199) (4228S), PI3K (4257S), AKT (4691S), p-AKT (Ser473) (9271S), ERK1/2 (9102S), p-ERK1/2 (Thr202/Tyr204) (9101S), mTOR (2972S), p-mTOR (Ser2448) (2971S), p-Raptor (Ser792) (2083S), Raptor (2280S), AMPKα (5832S), p-AMPKα (Thr172) (2535S), SREBP1 (95879S), p-ACC (Ser79) (11818S), ACC (3676S), p-STAT3 (Tyr705) (9131S), STAT3 (4904S), p-NF-κB p65 (Ser536) (3033S), and NF-κB p65 (4764S). Antibodies against Bcl-2 (sc-7382), Bax (sc-7480), and β-actin (sc-47778) were obtained from Santa Cruz Biotechnology (Santa Cruz, CA, USA). Horseradish peroxidase (HRP)-conjugated anti-mouse and anti-rabbit IgG secondary antibodies (7076 and 7074, respectively) were obtained from Cell Signaling Technology.

For SDS-PAGE, 10–20 μg of protein was loaded per lane. All primary antibodies were used at a dilution of 1:2000, except for the anti-β-actin antibody, which was used at 1:4000. HRP-conjugated secondary antibodies were used at a dilution of 1:5000. Immunoreactive bands were detected using the EZ-Western Lumi Pico Kit (DG-WP250; DoGenBio Co., Ltd., Seoul, Republic of Korea) and imaged with an Amersham Imager 600 (GE Healthcare Bio-Sciences AB, Uppsala, Sweden). Phosphoprotein signals were normalized to the corresponding total target protein, and other protein signals were normalized to β-actin. Western blot experiments were performed independently three times (*n* = 3). Band intensities were quantified using ImageJ software ver 1.0 (https://imagej.nih.gov/ij, accessed on 27 September 2021).

### 4.6. Intracellular ROS and Mitochondrial Membrane Potential Assays

Intracellular reactive oxygen species (ROS) levels were measured using 2′,7′-dichlorofluorescein diacetate (DCF-DA) staining followed by flow cytometric analysis, according to previously described protocols [34,35]. Mitochondrial membrane potential was assessed using the MitoProbe™ JC-1 Assay Kit (Invitrogen™, Waltham, MA, USA) according to the manufacturer’s instructions. Carbonyl cyanide *m*-chlorophenyl hydrazone (CCCP) was used as a positive control for mitochondrial membrane depolarization. Both the intracellular ROS assay and the mitochondrial membrane potential assay were performed in three independent experiments, with each treatment condition assessed in technical triplicate within each experiment.

### 4.7. Xenograft Study

Four-week-old male BALB/c nude mice were purchased from Junbiotech (Hwaseong, Republic of Korea) and housed in the specific pathogen-free (SPF) animal facility of Kyung Hee University Medical Center. Mice were housed 4–5 animals per cage at 22 ± 2 °C and 50 ± 10% relative humidity under a 12 h/12 h light/dark cycle, with sterilized chow and water available ad libitum. Wood-shaving bedding was provided in the cages. No additional environmental enrichment was provided. Animals were acclimated to the facility for one week before the experiment.

A total of 18 mice were used, with six mice allocated to each of three experimental groups (*n* = 6 per group), and the individual mouse was considered the experimental unit. The group size was prespecified in the approved animal study protocol, taking into account expected xenograft establishment, inter-animal variability in tumor growth, and the need to obtain sufficient tumor samples for subsequent molecular, histological, and metabolic analyses. No formal a priori sample-size or statistical power calculation was performed. Successful establishment of a palpable tumor before treatment allocation was required for inclusion in the treatment phase. Prespecified exclusion criteria included failure of initial tumor engraftment or non-treatment-related traumatic loss during animal husbandry. No animals or data points were excluded from the final analyses.

HCT116 cells (5 × 10^6^ cells) were mixed with Matrigel (Corning Inc., Corning, NY, USA) at a 1:1 ratio and subcutaneously injected into the right flank of each mouse on day 0. When tumors became palpable 3 days after tumor-cell inoculation, mice were allocated to the vehicle-control group or PSY-treatment groups receiving 50 or 100 mg/kg PSY using tumor-size-balanced allocation to achieve comparable baseline tumor burdens among the groups. No formal randomization sequence was generated. No blinding was used during treatment administration, outcome assessment, histological assessment, or data analysis; investigators were aware of group allocation. Standardized procedures were used for treatment administration, tumor measurement, histological assessment, and data analysis to minimize experimental variability.

Mice received vehicle (water) or PSY at 50 or 100 mg/kg by oral gavage at a volume of 200 μL per administration three times per week for 3 weeks. Tumor dimensions were measured using calipers, and tumor volume was calculated using the following formula: Tumor volume = length × width^2^ × 0.5. Body weight, tumor size, general appearance, and activity were monitored at least three times per week throughout the experimental period.

Predefined humane endpoints included unexplained body-weight loss exceeding 20%, complete loss of appetite for 24 h, impaired food or water intake associated with tumor necrosis, infection, or ulceration, persistent severe gastrointestinal abnormalities, prolonged lateral recumbency, severe neurological abnormalities, marked hypothermia, serious infection, or tumor burden exceeding 10% of normal body weight. Animals meeting any of these criteria were to be euthanized. No treatment-related adverse events occurred, and no animal reached a predefined humane endpoint during the study.

At the experimental endpoint, mice were euthanized by gradual-fill CO_2_ exposure in a dedicated chamber at a flow rate corresponding to 20% of the chamber volume per min. Death was confirmed before animals were removed from the chamber, and CO_2_ flow was maintained for at least 1 min after confirmation of death. Tumors were then excised, weighed, and collected for molecular, histological, and lipid analyses. Tumors from three mice per group were stored as frozen tissue for fatty-acid profiling, whereas tumors from the remaining three mice were paraffin-embedded for histological analysis. All animal procedures were approved by the Institutional Animal Care and Use Committee of Kyung Hee University Medical Center (KHMC-IACUC 23-009).

### 4.8. Immunohistochemistry

Xenograft tumor tissues were fixed in formalin, embedded in paraffin, and sectioned at a thickness of 5 μm. Sections were deparaffinized, rehydrated, and subjected to antigen retrieval using citrate buffer (pH 6.0). After blocking, sections were incubated overnight at 4 °C with primary antibodies against Ki-67 (GTX16667), cleaved caspase-3 (9661S), p-STAT3 (Tyr705) (9145S), COX2 (12282S), ATF4 (11815S), p-AKT (9271S), or p-ERK1/2 (9101S), as previously described [36,37].

The antibody against Ki67 was obtained from GeneTex Inc. (Irvine, CA, USA) and the remaining antibodies were purchased from Cell Signaling Technology. Primary antibodies were used at dilutions ranging from 1:100 to 1:200.

The sections were subsequently incubated with biotinylated secondary antibodies (Vector Laboratories, Newark, CA, USA) for 30 min, followed by HRP-conjugated streptavidin using an ABC Kit (Vector Laboratories) for 30 min. Immunoreactivity was visualized using 3,3′-diaminobenzidine (DAB), and the sections were counterstained with hematoxylin. Images were acquired using an Olympus BX53 microscope under comparable imaging conditions for all experimental groups.

DAB staining was quantified using the IHC Profiler plugin for ImageJ (https://sourceforge.net/projects/ihcprofiler/; accessed on 2 September 2026) [38]. Following color deconvolution, DAB pixels were classified as high positive, positive, low positive, or negative and assigned weights of 3, 2, 1, or 0, respectively. A pixel-based H-score was calculated as (3 × percentage of high-positive pixels) + (2 × percentage of positive pixels) + (1 × percentage of low-positive pixels), yielding a score from 0 to 300. Two to three fields per animal were analyzed and averaged to obtain one value per mouse. Three mice per group were included, and the mouse was the biological unit for statistical analysis.

### 4.9. Lipid Extraction and LC-MS/MS Analysis

Fatty-acid profiling was performed using three independent frozen xenograft tumor samples from each treatment group (*n* = 3) to evaluate the effect of PSY on tumor lipid metabolism. Approximately 100 mg of tumor tissue was homogenized on ice in 1 mL of methanol/chloroform (2:1, *v*/*v*). Lipids were extracted using a modified Bligh–Dyer method. Briefly, 200 μL of water was added to each homogenate, followed by vortex mixing and centrifugation at 13,500× *g* for 15 min at 4 °C. The lower organic phase was carefully collected, dried under a stream of nitrogen, and reconstituted in 100 μL of acetonitrile/methanol (7:3, *v*/*v*) before LC-MS/MS analysis.

Fatty-acid profiling was performed using a Vanquish UHPLC system (Thermo Fisher Scientific, Waltham, MA, USA) coupled to a TripleTOF 5600+ MS (SCIEX, Foster City, CA, USA). Chromatographic separation was achieved using an ACQUITY UPLC CSH C18 column (2.1 × 100 mm, 1.7 μm; Waters Corp., Milford, MA, USA). The mobile phase consisted of solvent A (acetonitrile/water, 60:40, *v*/*v*) and solvent B (isopropanol/acetonitrile, 90:10, *v*/*v*), both containing 10 mM ammonium formate and 0.1% formic acid. The flow rate was 0.3 mL/min, and the injection volume was 5 μL. MS analysis was performed in negative electrospray ionization mode using a source temperature of 400 °C and an ion-spray voltage of –4500 V.

Fatty acids were identified by comparing their retention times and MS/MS spectra with those of authentic standards and spectral-library data. Authentic standards for arachidonic acid, hexacosanoic acid, myristic acid, oleic acid, stearic acid, palmitoleic acid, and linoleic acid were obtained from Sigma-Aldrich (St. Louis, MO, USA) and used to support compound identification and relative quantification. Integrated peak areas were used for relative abundance comparisons. Peak integration and data processing were performed using SCIEX Analyst TF version 1.7 and PeakView version 2.2.

### 4.10. Cell Viability Assay

Cell viability was assessed by the MTT assay after 72 h of treatment, as described previously [39]. For drug-combination experiments, HCT116 cells were treated with PSY (0–200 μg/mL) together with 5-fluorouracil (5-FU, 0–20 μM), SN38 (0–20 nM), or oxaliplatin (0–8 μM). Additional PSY–5-FU combination matrices were evaluated in HT-29 and SW480 cells using PSY concentrations of 0–200 μg/mL and 5-FU concentrations of 0–35 μM and 0–70 μM, respectively. Three independent experiments were performed, each with triplicate wells per treatment condition. Technical-well values were averaged within each experiment, and viability data are presented as mean ± standard error of the mean (SEM) across the three independent experiments. Fraction affected (Fa) was calculated as 1 minus relative viability expressed as a fraction of the vehicle-control value. CI values were calculated with CompuSyn ver. 1.0 (ComboSyn Inc., Paramus, NJ, USA) according to the Chou–Talalay method [40], using the mean viability across the three experiments. CI < 1, CI = 1, and CI > 1 indicate synergistic, additive, and antagonistic interactions, respectively. Each CI value is a point estimate derived from the averaged viability data; replicate-specific CI values and their SEM were not calculated.

To complement the MTT-based measurements, direct cell counting by trypan blue exclusion using the LUNA-FL automated cell counter was performed in HCT116 and SW480 cells after 72 h of treatment. HCT116 cells were treated with PSY (25 μg/mL) and/or 5-FU (5 μM), whereas SW480 cells were treated with PSY (12.5 μg/mL) and/or 5-FU (8.75 μM). Viable cell counts were expressed as percentages of the corresponding vehicle-control count within each independent experiment. Three independent experiments were performed, with one well per treatment condition in each experiment.

Cell-free controls containing vehicle, PSY, 5-FU, or their combination were included to assess potential treatment-related interference with the MTT readout. MTT assay absorbance was measured at 570 nm in both HCT116 cell-containing wells and cell-free wells for each treatment condition.

### 4.11. Cell-Cycle and Apoptosis Analysis

For cell-cycle analysis, HCT116 cells were treated for 48 h, harvested, fixed in 70% ethanol, and stained with propidium iodide (PI). Cellular DNA content was measured using an FACS Canto II flow cytometer (BD Biosciences, Franklin Lakes, NJ, USA), and the proportions of cells in the sub-G_1_, G_0_/G_1_, S, and G_2_/M phases were determined [41].

Apoptosis was assessed using an Annexin V-FITC/PI dual-staining kit (ApoScan Annexin V FITC Apoptosis Detection Kit, BioBud, Seoul, Republic of Korea), as previously described [41]. After 48 h of treatment, cells were harvested, stained according to the manufacturer’s instructions, and analyzed by flow cytometry [35]. The percentage of Annexin V-positive cells, comprising both early and late apoptotic populations, was quantified. Data were obtained from three independent experiments.

### 4.12. Statistical Analysis

Data are presented as mean ± SEM unless otherwise indicated. Biological replication comprised two samples per group for RNA sequencing, six mice per group for the xenograft growth study, three tumors per group for IHC and fatty-acid profiling, and three independent experiments for Western blot and drug-combination viability analyses. Technical replicates were averaged within each biological replicate before statistical analysis. Exact biological replicate numbers for the other assays are stated in the corresponding figure legends. RNA-seq analyses and multiple-testing correction are described in Section 4.3. Statistical analyses were conducted using GraphPad Prism 9.5.1 (GraphPad Software, La Jolla, CA, USA). Comparisons between two groups were performed using Student’s *t*-test, whereas comparisons among three or more groups were performed using one-way analysis of variance (ANOVA) followed by Tukey’s multiple-comparisons test, unless otherwise indicated. For the xenograft study, body-weight and tumor-volume data were analyzed by two-way repeated-measures ANOVA, with treatment and time as factors, followed by Tukey’s multiple-comparisons test. Final tumor weights were analyzed by one-way ANOVA followed by Tukey’s multiple-comparisons test. IHC H-scores are presented as mean ± SEM for three mice per group. For each marker, the three treatment groups were compared using one-way ANOVA followed by Tukey’s multiple-comparisons test, including comparisons between the two PSY doses. No formal testing of statistical assumptions, such as normality or sphericity, was prespecified or performed. A *p* value of <0.05 was considered statistically significant.

## 5. Conclusions

PSY reduced HCT116 xenograft growth and was associated with changes in cellular stress responses, survival-signaling markers, and lipid-related measurements. In vitro combination analysis supported synergistic interactions with 5-FU at multiple tested concentration pairs, accompanied by enhanced apoptosis and changes in signaling markers. These findings support further preclinical investigation of PSY and its combination with 5-FU. Functional pathway studies, in vivo combination testing, and further extract characterization are needed to establish mechanisms and broader applicability.

## Figures and Tables

**Table 1:** Combination index (CI) and fraction affected (Fa) values for PSY combined with chemotherapeutic agents in HCT116 cells after 72 h. Fa was calculated as 1 minus relative viability, expressed as a fraction of the vehicle-control value. CI values were calculated from mean viability across three independent experiments, each containing triplicate wells per condition. CI < 1, CI = 1, and CI > 1 indicate synergistic, additive, and antagonistic interactions, respectively. CI values are point estimates without replicate-based SEM or confidence intervals.

			5-Fluorouracil (μM)					SN38 (nM)					Oxaliplatin (μM)				
			1.25	2.5	5	10	20	1.25	2.5	5	10	20	0.5	1	2	4	8
PSY (μg/mL)	12.5	Fa	0.165	0.322	0.428	0.554	0.677	0.257	0.353	0.432	0.552	0.675	0.234	0.369	0.556	0.597	0.655
		CI	1.062	0.75	0.811	0.888	0.998	1.280	1.175	1.290	1.159	0.988	2.027	1.439	0.876	1.228	1.578
	25	Fa	0.273	0.425	0.503	0.592	0.681	0.297	0.393	0.506	0.639	0.744	0.190	0.299	0.501	0.628	0.659
		CI	0.951	0.717	0.577	0.897	1.079	1.304	1.187	1.053	0.846	0.727	3.428	2.592	1.402	1.173	1.680
	50	Fa	0.392	0.460	0.496	0.597	0.720	0.370	0.390	0.496	0.639	0.785	0.308	0.302	0.470	0.620	0.672
		CI	1.075	1.010	0.819	1.133	1.082	1.456	1.709	1.509	1.143	0.753	2.255	3.242	2.102	1.544	1.825
	100	Fa	0.508	0.572	0.692	0.788	0.848	0.689	0.736	0.765	0.829	0.886	0.638	0.642	0.593	0.680	0.766
		CI	1.409	1.258	0.662	0.791	0.751	1.105	1.004	0.968	0.812	0.653	1.313	1.396	1.901	1.685	1.446
	200	Fa	0.884	0.898	0.913	0.914	0.926	0.931	0.935	0.930	0.936	0.935	0.922	0.924	0.923	0.928	0.924
		CI	0.663	0.614	0.561	0.607	0.619	0.826	0.801	0.841	0.809	0.835	0.788	0.793	0.796	0.779	0.847

**Table 2:** Primer sequences used for quantitative real-time PCR. 4EBP1, *SREBP1*, and *ACC* denote *EIF4EBP1*, *SREBF1*, and *ACACA*, respectively.

Gene Name	Primer Sequence (5′ to 3′)	
	Forward	Reverse
*ATF4*	TTCTCCAGCGACAAGGCTAAGG	CTCCAACATCCAATCTGTCCCG
*DDIT3*	GGTATGAGGACCTGCAAGAGGT	CTTGTGACCTCTGCTGGTTCTG
*DDIT4*	GTTTGACCGCTCCACGAGCCT	GCACACAAGTGTTCATCCTCAGG
*ASNS*	CTGTGAAGAACAACCTCAGGATC	AACAGAGTGGCAGCAACCAAGC
*TRIB3*	GCTTTGTCTTCGCTGACCGTGA	CTGAGTATCTCAGGTCCCACGT
*CHAC1*	GTGGTGACGCTCCTTGAAGATC	GAAGGTGACCTCCTTGGTATCG
*DUSP5*	GCCCGCGGGTCTACTTCCTCAAA	ATTTCAACTGGGCCACCCTGG
*CARS1*	CCATGCAGACTCCACCTTTAC	GCAATACCACGTCACCTTTTTC
*STC2*	GCATGACTTTTCTGCACAACGCT	GGCTTATGCAGCCGAACCTGTG
*SESN2*	AGATGGAGAGCCGCTTTGAGCT	CCGAGTGAAGTCCTCATATCCG
*PCK2*	TAGTGCCTGTGGCAAGACCAAC	GAAGCCGTTCTCAGGGTTGATG
*4EBP1*	CACCAGCCCTTCCAGTGATGAG	CCTTGGTAGTGCTCCACACGAT
*DHCR7*	TCCACAGCCATGTGACCAATGC	CGAAGTGGTCATGGCAGATGTC
*DHCR24*	CAGGAGAACCACTTCGTGGAAG	CCACATGCTTAAAGAACCACGGC
*FDFT1*	GGTCCCGCTGTTACACAACT	AAAACTCTGCCATCCCAATG
*LSS*	GACGACCGATTCACCAAGAGCA	AGACATGCTCCTGGAAGGCAGT
*PPARG*	AGCCTGCGAAAGCCTTTTGGTG	GGCTTCACATTCAGCAAACCTGG
*SREBP1*	ACTTCTGGAGGCATCGCAAGCA	AGGTTCCAGAGGAGGCTACAAG
*FASN*	TTCTACGGCTCCACGCTCTTCC	GAAGAGTCTTCGTCAGCCAGGA
*ACC*	TTCACTCCACCTTGTCAGCGGA	GTCAGAGAAGCAGCCCATCACT
*GAPDH*	CCCATCACCATCTTCCAGGAG	GTTGTCATGGATGACCTTGGCC

## Data Availability

The RNA-sequencing dataset generated in this study is publicly available in the NCBI Gene Expression Omnibus under accession number GSE285901. Complete gene-level results from the original and complementary RNA-seq analyses are provided in Supplementary Table S1. Other data supporting the findings of this study are available from the corresponding author upon reasonable request.

## Supplementary Materials

The following supporting information can be downloaded at: https://www.mdpi.com/article/10.3390/ijms27209238/s1.

## Abbreviations

The following abbreviations are used in this manuscript:

Abbreviation	Definition
CRC	Colorectal cancer
5-FU	5-Fluorouracil
ER	Endoplasmic reticulum
UPR	Unfolded protein response
ROS	Reactive oxygen species
DEG	Differentially expressed gene
PPI	Protein–protein interaction
GO	Gene Ontology
KEGG	Kyoto Encyclopedia of Genes and Genomes
XIC	Extracted ion chromatogram
NCBI	National Center for Biotechnology Information
GEO	Gene Expression Omnibus
qRT-PCR	Quantitative real-time polymerase chain reaction
DMSO	Dimethyl sulfoxide
SDS-PAGE	Sodium dodecyl sulfate–polyacrylamide gel electrophoresis
HRP	Horseradish peroxidase
DCF-DA	2′,7′-Dichlorofluorescein diacetate
CCCP	Carbonyl cyanide *m*-chlorophenyl hydrazone
LC-MS/MS	Liquid chromatography–tandem mass spectrometry
UHPLC	Ultra-high-performance liquid chromatography
CI	Combination index
Fa	Fraction affected
PI	Propidium iodide
FITC	Fluorescein isothiocyanate
SEM	Standard error of the mean
BH	Benjamini–Hochberg
